# Investigating the value of balance and proprioception scores to predict lower limb injuries in professional judokas

**DOI:** 10.1038/s41598-023-49114-0

**Published:** 2023-12-08

**Authors:** Shirzad Mian Darbandi, Mostafa Zarei, Haniyeh Mohammadi, Mahdi Hosseinzadeh

**Affiliations:** 1 Department of Sport Rehabilitation and Health, Faculty of Sports Sciences and Health, Shahid Beheshti University, Tehran, Iran; 2 Department of Sport Injuries and Corrective Exercises, Faculty of Physical Education and Sports Sciences, Shomal University, Amol, Iran; 3 Department of Sport Injuries and Corrective Exercises, Sport Sciences Research Institute, No. 3, 5th Alley, Miremad Street, Motahhari Street, PO Box: 1587958711, Tehran, Iran

**Keywords:** Health care, Risk factors

## Abstract

We investigated the ability of balance and proprioception screening tests to predict lower limb injuries in professional male judokas. Fifty-three male judokas of the national teams (Mean ± SD, age 18.68 ± 3.08 years, weight 75.34 ± 11.62 kg, height 175.28 ± 7.24 cm) participated in this study. Limits of stability (LOS), single leg stability (SLS), and knee joint position sense (JPS) was recorded as the screening tests before starting the 10 month follow up. Lower limb injury was recorded all through the follow up. Fifteen lower limb sport injuries were recorded for 53 judokas during the follow-up recordings. Significant accuracy of SLS, overall bilateral ratio (AUC 0.646, 95% CI 0.452–0.839, *p* = 0.046), as well as JPS 60° bilateral ratio (AUC 0.657, 95% CI 0.480–0.834, *p* = 0.044), and LOS overall (AUC 0.696, 95% CI 0.551–0.840, *p* = 0.031) were revealed discriminating between injured and uninjured judokas. The optimum cut-off of SLS, overall bilateral ratio, JPS 60° bilateral ratio, and LOS overall associated with belonging to uninjured judokas group was ≤ 1.15%, ≤ 1.09%, and ≤ 1.09 respectively (sensitivity, 0.763, 0.711, 0.789 respectively; specificity, 0.600). Although the absolute unilateral balance and proprioception scores were almost the same between injured and non-injured judokas, the bilateral ratio of both these indices were different between the two groups. Lower limbs bilateral balance and proprioception asymmetries is a more important risk factor than the absolute unilateral balance and proprioception scores for sustaining lower limb injuries in professional male judokas. Medical professionals and coaches are suggested to use these findings as pre-participation screening tools identify injury-prone athletes.

## Introduction

Judo as one of the most recognized martial arts is practiced in more than 150 countries^1^. This Olympic sport of course leads to injuries though. According to recent reports, the overall injury rate during high-level European judo competitions (between years 2005 and 2020) was reported 2.5%^2,3^. Generally, 30% of judo injuries occur in the lower limbs, it is therefore essential to identify risk factors and prevent lower limb injuries in this sport^2,3^. The fight in judo competition starts with both players standing and trying to knock each other off balance^4^. The overall performance of the body in judo, like most of other sports, increases therefore by improving the postural control^5,6^. Postural control is consisted of two components: postural orientation and postural stability^7^. The judokas ability to appropriately control their posture is further essential in order to avoid injuries of the lower limbs^8^. Proprioception furthermore, which is defined as the ability of an individual to integrate sensory signals from different mechanical receptors to regulate the position of the body and movements in space, has also an important role in controlling the movement and balance^9–11^. Deficits of proprioception, including kinesthesia and joint position sense (JPS), exist in patients with chronic ankle instability when compared with the uninjured contralateral lower limb and healthy people^12,13^. Lower limb JPS tests appear to have appropriate validity in distinguishing injured knees from asymptomatic knees^13,14^. Further evidence of high methodologic quality is however required to determine the reliability, responsiveness, and applicability of JPS to develop standardized evidence-based screening tools for predicting lower limb injuries^12–14^.

Whereas postural orientation is defined as the capacity to sustain proper connection between body segments and further the relationship between the body and the surroundings in order to perform a certain task, postural stability as a fundamental contributing factor of functional movement, is the ability to control the body position in space to move smoothly and keep the balance^7^. It has been very well revealed that postural stability is diminished in people with lower limb injury. Furthermore, sway magnitude during single leg stance (SLS) was augmented significantly in the lower limb injured group rather than the healthy individuals^15^. In a prospective study it was further found that female athletes who experienced a diminution in postural stability during the single-leg stance phase are more eligible for sustaining non-contact lower limb injury^16^. Moreover, the limit of stability test (LOS) also allows measurable variables of dynamic stability to be acquired in a clinically manageable manner due to its portability.

The LOS test is a reliable test to evaluate postural stability in adolescents^17,18^. The postural stability is immature and/or impaired at the two ends of the lifespan (in young children and older adults)^19,20^. Adolescents, however, have an improved postural stability and have the ability to develop their LOS, which reduces their risk of falls^21^. Adolescents participating athletics activity actually depend on their LOS to keep their balance up while performing dynamic movements such as throwing, catching, kicking, and blocking^21^. It is further demonstrated that injury to lower limb ligaments weakens LOS due to swelling, pain, and instability. It is demonstrated that male gymnasts who have sustained more lower limb injuries recorded significantly higher values in reaction time and lower values in movement velocity during LOS test^22^. Several other researches have however denied this claim and did not report the screening balance tools as suitable predictors for lower limb injury^23,24^. A functional inert stability is although necessary to prevent injury^20^, but definitions and objective measures to be used as screening balance tools are lacking^20,25^. Developing injury prevention programs in athletics activity is very important in order to reduce injuries^26^. The use of appropriate pre-sessional screening tools to identify injury-prone athletes is also very important in developing injury prevention programs^20,27,28^. Screening for the balance and proprioceptive ability of those athletes who participate in long-term training in the same sport is highlighted in previous studies specifically in order to acquire practical injury prevention strategies^11,20^. Due to the lack of specific information in this regards on judokas, the purpose of this study was to investigate the ability of balance and proprioceptive screening tests to predict lower limb injuries in male professional judo players.

## Methods

### Participants

The protocol of this prospective cohort study was approved by the institutional research committee at Sport Sciences Research Institute of Iran. Fifty-three male judokas of the national teams voluntarily participated in the pre-participation screening tests, after the necessary coordination/approval of the national judo federation and the coaches of the national teams. Participants had to be a member of one of the Iranian national judo teams (one of the age level cadet, junior, senior), and be training full-time in a high-level competition environment (at least 5 practices per week). A formal sample size calculation was not performed because all members of the national teams in Iran were recruited (n = 53).

### Ethical considerations

The study was conducted according to the principles of the Declaration of Helsinki and its latest amendments, and the protocol of the study was approved by the research ethics committee of sport sciences research institute of Iran, registration number IR.SSRC.1400.067. An informed consent was obtained from all judokas participants of this study and/or their legal guardians.

### Procedures

Information regards the athletes’ training experience age, height, weight, BMI, top guard and injury sustaining history were collected. Judokas then took part in both dynamic stability and the proprioception tests. The LOS, and SLS were recorded using the Biodex Balance System, and the JPS proprioception test at 60˚ of knee flexion was recorded using the Biodex Isokinetic pro 4 system. All the pre-participatory tests were carried out from 8.00 to 12.00 o’clock at the sports medicine laboratory of Shahid Beheshti University of Iran during June–July 2020. Then the judokas’ lower limb injury was recorded all through the 10 month follow up period while participating in the judo national team camps in 2020–2021.

### Biodex balance system (BBS)

BBS (Biodex Medical System Inc., Shirley, New York) test formats include postural stability (PS), limits of stability (LOS), single leg stability (SLS), and fall risk stability (FRS). SLS and FRS tests can be compared with normative data, as well as PS and LOS tests in different difficulty levels^29^. Bilateral reports (comparison of PS performance standing on one leg vs. standing on the other leg) are available in most PS test options. However, due to the inherent nature of judo in the frequent use of single-leg techniques by judokas, the SLS and LOS tests were preferably recorded in this study.

### Single leg stability test (SLS)

Prior to data acquisition, the athlete was asked to center the foot on the platform in a position that was level and stable. This foot placement procedure was maintained the same throughout all 3 trials for the test leg. This position was used as the level reference point from which degree of displacement was measured. The athlete was instructed to stand on 1 foot with the knee slightly flexed on the free-moving stability platform, with the contralateral knee flexed. The judoka was then instructed to keep the platform as stable as possible^29^.

Considering the researchers experience with testing on level 4 of the BSS, level mean the countdown 5-3 was selected for all testing^30^. The judokas were instructed to cross their arms at their chest to minimize their use in attaining balance, as per system operation procedures. The stability system was positioned facing the corner of the room; no verbal feedback was provided during the test and each athlete was asked to look straight ahead and focus on a point on the plate monitor in front of them. Each leg was tested 3 times, utilizing the BSS^29^. The mean displacement from the referenced, level position during the 20-s trial was calculated for each trial. The mean and standard deviation of the 3 trials was calculated by the stability system. The data were analyzed and reported as total stability index, AP stability index, and ML stability index, which is the mean displacement of the platform in degrees, from a level position.

### Limit of stability test (LOS)

BBS was again used to measure displacement of LOS. LOS is composed of a circular platform that allows 0–20° of platform surface tilt in a 360° range of motion. LOS test involves 12 dynamic stability levels that ranges from 1 (most unstable) to 12 (most stable). The LOS consists of standing on the platform and leaning in eight directions to make a cursor displayed on the system’s screen hit a target. Level 4 was selected for all testing of LOS.

The LOS test challenges athletes; the participant must simultaneously maintain their center of gravity and perform the unpredictable balancing tasks of the BBS on an unstable platform. During each test, athlete must shift their weight to move the cursor from the center target to a blinking target and back as quickly and with as little deviation as possible. The same process was repeated for each of nine targets. Higher scores indicated better performance. Athletes were allowed to hold their arms comfortably and adopt their desired strategy for hitting the targets. They were not allowed to grasp the handles to recover balance; Therefore, if judokas were unable to complete the task without holding onto the handles, the test was terminated^31^. The mean and standard deviation of the 3 trials was calculated by the stability system. The data were analyzed and reported as total LOS, forward, backward, rightward, leftward, forward-rightward, forward-leftward, backward–rightward, and backward-leftward stability index, which is the mean displacement of the platform in degrees, from a level position.

### Proprioception tests

#### Knee joint position sense (JPS)

To assess joint position sense, the Biodex Isokinetic pro 4 system (Con-Trex, CMVAG, Zürich, Switzerland) was used. The reliability and validity of this dynamometer has been described to be excellent ((ICC) = 0.99–1.00)^32^. Before each test session, the dynamometer was set in accordance with the manufacturer’s recommendations. Judokas performed a standardized few-minute general warm-up on a Monark cycling ergometer at a moderate pace. Tests were conducted in a standardized sitting position. Athletes completed two or three smooth, continuous repetitions of each motion just before the respective test, to familiarize themselves with the apparatus and movement, and to promote relaxation. To prevent unwanted movements, players were fixed with straps across the shoulders, chest, and hip; the cuff of the dynamometer’s lever arm was attached proximal to the malleoli of the ankle. The rotational axis of the knee joint was aligned with the dynamometer’s rotational axis. The testing was performed on both the dominant leg and the non-dominant leg.^33,34^.

The knee joint proprioception test was examined at the target flexion angle of 60° with the active movement of the athlete in the BBS. To memorize the target angle, the participant’s leg was passively moved to the target angle, the knee was held in the target position for five seconds, and then the subject was asked to return the knee actively back to the test position (angle of 90° was considered as starting position for the target angle of 60°).

The participant was asked to extend their knee toward the previously selected target angle. They were instructed to press the stop button when the memorized target angle was reproduced. The tests were performed three times with closed eyes for target angle, with a 30-s rest period between repetitions.

The score obtained from the proprioception measurement was the deviation from the referenced joint position and was recorded as an absolute error in degrees. The mean joint positioning error of the three measurements of joint position was used for analysis. A lower mean error value indicated the better proprioception performance^35^.

### Injury data collection

The injury data collection of judokas during the 10 months of research were followed and recorded. The whole procedures have already been explained elsewhere^27^. The definition of various types of injury and the procedure for the injury data collection was explained to all of the team medical staff and coach representatives. All the injury data was recorded by the team physiotherapists and athletic trainers, and was collected weekly.

### Lower limb injury definition

A lower limb injury was defined in accordance with established consensus statements as any musculoskeletal injury or acute pain in the lower limbs leading to an immediate cessation of match play or training sessions and for which the judokas required a consultation with a health care practitioner^36^. The injury was usually later confirmed by the national team medical staffs through clinical examination. Moderate and/or sever injuries (expected day-loss greater than 2 days) were checked by orthopedics specialists, who diagnosed these injuries using clinical tests and medical imaging.

### Statistical analysis

The data is shown as average ± standard deviation (SD) with 95% confidence intervals for the average differences. The research data related to the demographic characteristics of athletes and research variables were analyzed in two sections of descriptive and inferential statistics with SPSS for Windows, version 26.0, (SPSS Inc., Chicago, pernios, USA software). The Shapiro–Wilk statistical test assessed the normality of the data. Two group comparisons (injured vs. uninjured judokas) were conducted using the Student t test. Logistic regression analysis was then used to create a separate model with each of the variables of the balance and proprioception. The *p* value was set at ≤ 0.05. The corresponding receiver operating curve (ROC) was plotted for defining a cut off for the indicators of balance and proprioception of the professional Judokas of this study.

## Results

The professional national level judokas of this study were followed up to a mean of 40 ± 4 weeks during participation in the national team camps in 2020–2021. Fifteen lower limb sport injuries were recorded for 53 judokas during this period. Eleven injuries occurred in the dominant lower limb and 4 occurred in the non-dominant lower limb. Age, height, weight, BMI, SLS over left lower limb, LOS in forward, backward, and leftward directions, and unilateral KJP scores did not differ significantly between injured and uninjured judokas (Tables 1 and 2: all *p* ˃ 0.05) while the bilateral ratio of both the SLS and KJP were significantly smaller in uninjured compared to injured judokas (Table 2: all *p* < 0.05).

**Table 1:** The bio-sociodemographic information of the total sample study population and the results of the statistical comparisons between Judokas with or without lower limb injury.

Bio-sociodemographic	Total sample (n = 53, 100%)	With lower limb injury (n = 15, 28%)	Without lower limb injury (n = 38, 72%)	*p*
Age level (Category; M 1;3)	2 (1;3)	2 (1;3)	2 (1;3)	0.917
Cadet (n, %)	17 (32.1%)	5 (33.3%)	12 (31.6%)	
Junior (n, %)	18 (34%)	5 (33.3%)	13 (34.2%)	
Senior (n, %)	18 (34%)	5 (33.3%)	13 (34.2%)	
Age (years, Mean ± SD)	18.68 ± 3.08	18.73 ± 2.96	18.66 ± 3.16	0.937
Weight (kg, Mean ± SD)	75.34 ± 11.62	76.33 ± 13.77	74.95 ± 10.84	0.700
Height (cm, Mean ± SD)	175.28 ± 7.24	177.40 ± 8.01	174.45 ± 6.85	0.184
BMI (kg m^−2^, Mean ± SD)	23.09 ± 3.66	22.54 ± 4.21	23.31 ± 3.45	0.498

*kg* kilogram, *SD* standard deviation, *cm* centimeter, *m* meter.

**Table 2:** The dynamic balance, and proprioception information of the total sample study population and the results of the statistical comparisons between Judokas with or without lower limb injury.

Dynamic balance, proprioception	Total sample (n = 53, 100%)	With lower limb injury (n = 15, 28%)	Without lower limb injury (n = 38, 72%)	*p*
SLS, AP, right lower limb	2.06 ± 0.91	2.46 ± 1.19	1.91 ± 0.73	0.048
SLS, ML, right lower limb	1.45 ± 0.54	1.72 ± 0.65	1.34 ± 0.46	0.022
SLS, overall, right lower limb	2.82 ± 1.14	3.38 ± 1.49	2.60 ± 0.90	0.024
SLS, AP, left lower limb	1.98 ± 0.88	2.10 ± 1.22	1.94 ± 0.72	0.546
SLS, ML, Left lower limb	1.41 ± 0.57	1.50 ± 0.82	1.37 ± 0.45	0.497
SLS, overall, left lower limb	2.69 ± 1.16	2.90 ± 1.73	2.61 ± 0.86	0.433
SLS, ML bilateral ratio	1.14 ± 0.55	1.45 ± 0.84	1.02 ± 0.32	0.009
SLS, AP bilateral ratio	1.24 ± 0.99	1.74 ± 1.67	1.04 ± 0.41	0.020
SLS, overall bilateral ratio	1.19 ± 0.75	1.59 ± 1.23	1.03 ± 0.35	0.013
LOS forward	15.85 ± 9.63	13.87 ± 8.31	16.63 ± 10.10	0.352
LOS Backward	16.36 ± 11.15	16.33 ± 11.93	16.37 ± 11.00	0.992
LOS right	12.40 ± 7.31	8.07 ± 4.43	14.11 ± 7.56	0.006
LOS left	14.43 ± 7.25	13.53 ± 3.75	14.79 ± 8.25	0.575
LOS overall	10.81 ± 5.40	8.13 ± 3.20	11.87 ± 5.75	0.022
KJP right lower limb 60°	4.41 ± 2.16	4.86 ± 2.75	4.23 ± 1.90	0.349
KJP left lower limb 60°	4.31 ± 2.33	3.58 ± 2.15	4.61 ± 2.36	0.149
KJP 60° bilateral ratio	1.34 ± 1.15	1.92 ± 1.67	1.11 ± 0.78	0.021

*SLS* single leg stability, *AP* anteroposterior direction, *ML* mediolateral direction, *KJP* knee joint position, *LOS* limits of stability.

Correlation between the lower limb injury incidence and indicators of balance and proprioception indicators can be seen in Table 3. A significant correlation was observed between lower limb injury incidence and the indicators of balance and proprioception including SLS, ML bilateral ratio, SLS, AP bilateral ratio, SLS, Overall bilateral ratio, LOS Overall, and KJP 60˚ bilateral ratio.

**Table 3:** Correlations between lower limb injury incidence and Indicators of balance and proprioception (n = 53).

	Age (year)	BMI (kg m^−2^)	SLS, ML bilateral ratio	SLS, AP bilateral ratio	SLS, overall bilateral ratio	LOS overall	KJP 60° bilateral ratio
r	0.011	-0.095	0.357	0.319	0.340	0.314	0.317
p	0.937	0.498	0.009	0.020	0.013	0.022	0.021

*r* correlation coefficient, *p* significant (2-tailed), *BMI* body mass index, *SLS* single leg stability, *AP* anteroposterior direction, *ML* mediolateral direction, *KJP* knee joint position, *LOS* limits of stability.

The statistically significant overall models when compared to the null models for SLS, and KJP bilateral ratios, and LOS overall scores were provided in Table 4. These models generally explained 12–16% of the variations of the lower limb injury (meaning that lower limb injury can be explained by these full models), suggesting that predictions by these balance and proprioception indicators are fairly reliable. Furthermore, 67.9–77.4% of the judokas were correctly classified as with lower limb injured using the models provided by these indicators which is a large improvement.

**Table 4:** Significance of risk factors for lower limb injury in the logistic regression creating a separate model with each of the variables of the balance and proprioception.

	Coefficient (B)	*p*-value	R2 (Nagelkerke)	Odds ratios [Exp(B)]	95% Confidence Interval for Exp(B)	Overall model; overall predicted percentage
Age (years)	0.008	0.935	–	1.008	0.829–1.226	–
BMI (kg m^−2^)	− 0.062	0.491	–	0.940	0.787–1.122	–
SLS, ML bilateral ratio	1.474	0.026	0.165	4.365	1.198–15.902	X^2^(1) = 6.482, *p* ˂ 0.011; 75.5%
SLS, AP bilateral ratio	0.802	0.071	0.134	2.230	0.934–5.325	X^2^(1) = 5.200, *p* ˂ 0.023; 77.4%
SLS, overall bilateral ratio	1.088	0.046	0.151	2.968	1.019–8.647	X^2^(1) = 5.883, *p* ˂ 0.015; 77.4%
LOS overall	− 0.172	0.031	0.157	0.842	0.720–0.984	X^2^(1) = 6.134, *p* ˂ 0.013; 67.9%
KJP 60° bilateral ratio	0.596	0.044	0.128	1.816	1.017–3.241	X^2^(1) = 4.959, *p* ˂ 0.026; 73.6%

*BMI* body mass index, *SLS* single leg stability, *AP* anteroposterior direction, *ML* mediolateral direction, *KJP* knee joint position, *LOS* limits of stability.

**Figure 1:**
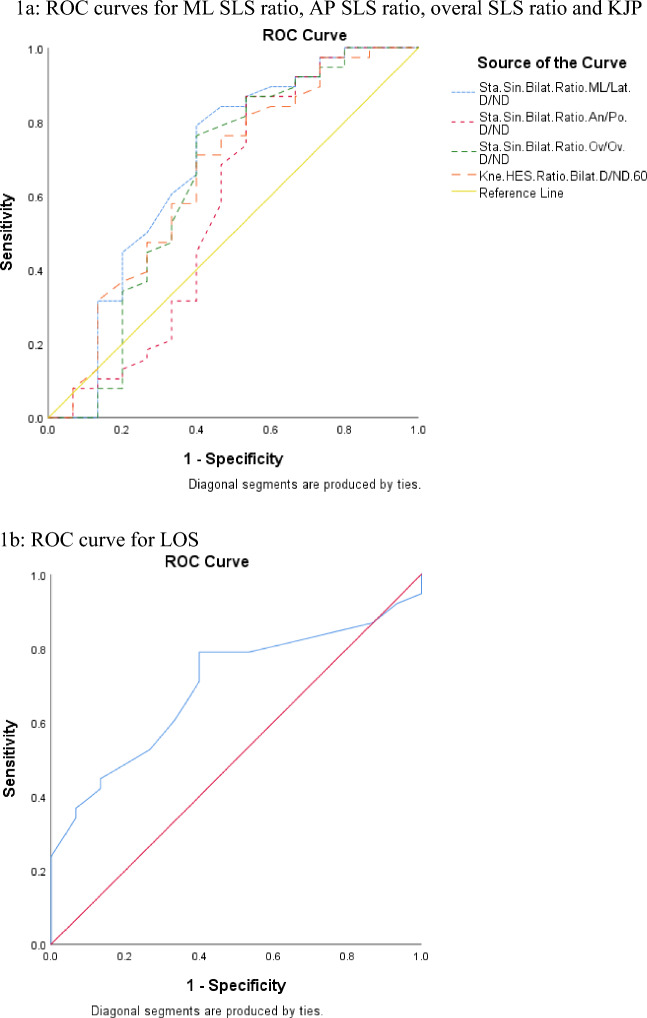
Receiver operating characteristic (ROC) curve for the indicators of balance and proprioception of the professional Judokas of this study. (**a**) ROC curves for ML SLS ratio, AP SLS ratio, overal SLS ratio and KJP. (**b**) ROC curve for LOS. The straight line shows the reference line, which was approximated by the ROC curve plotted on sensitivity (true positive rate) over 1-specificity (false positive rate) for the Indicators of balance and proprioception. Coordinates of the ROC curve and the optimal cut off point of these Indicators of balance and proprioception for the professional Judokas of this study are shown in Table 5.

Finally, predictive performance of balance and proprioception scores for lower limb injury in judokas, and ROC analyses showing significant accuracy of the balance and proprioception indicators, of this study, in discriminating between injured and uninjured judokas can be seen in Table 5 and Fig. 1, in which the duly optimum cut-off level of bilateral ML SLS, AP SLS, overall SLS and KJP ratio (Fig. 1a), and LOS score (Fig. 1b) associated with uninjured judokas of this study was provided.

**Table 5:** Predictive performance of balance and proprioception scores for lower limb injury in Judokas (n = 53).

Variables	Cutoff	Sensitivity (%)	Specificity (%)	AUC	AUC 95% CI
SLS, ML bilateral ratio	≤ 1.26	0.789	0.600	0.687	0.505–0.869
SLS, AP bilateral ratio	≤ 1.16	0.684	0.533	0.580	0.377–0.783
SLS, Overall bilateral ratio	≤ 1.15	0.763	0.600	0.646	0.452–0.839
LOS Overall	≥ 7.5	0.789	0.600	0.696	0.551–0.840
KJP 60° bilateral ratio	≤ 1.09	0.711	0.600	0.657	0.480–0.834

*AUC* area under curve, *CI* Confidence interval, *SLS* single leg stability, *AP* anteroposterior direction, *ML* mediolateral direction, *KJP* knee joint position, *LOS* limits of stability.

## Discussion

The aim of this study was to investigate the ability of balance and proprioception screening tests to predict lower limb injuries in professional male judokas during a 10-month follow-up. The main findings of this study was that although the absolute unilateral balance and proprioception scores were almost the same between injured and non-injured judokas (*p* ˃ 0.01), the bilateral ratio of both these indices were different between the two groups (*p* ≤ 0.01). To the best of the authors’ knowledge this finding for the first time demonstrates that lower limbs bilateral balance and proprioception asymmetries is a more important risk factor than the absolute unilateral balance and proprioception scores for sustaining lower limb injuries in professional judokas. Additionally, the logistic regression model indicated that all the bilateral ML, SLS ratio, bilateral AP, SLS ratio, bilateral overall SLS ratio, bilateral 60° KJP ratio and the overall LOS represented as important protective factors for lower limb injuries in professional judokas. When pre-participation results were compared between injured and uninjured judokas, it was shown that injured judokas had a significantly higher bilateral balance ratio (both the SLS and KJP ratio) accompanied by significantly lower overall LOS. Furthermore, the ROC curve demonstrated optimal cut off points of ≤ 1.26%, ≤ 1.16%, ≤ 1.15%, ≤ 1.09%, and ≥ 7.5 respectively for SLS, ML bilateral ratio, SLS, AP bilateral ratio, SLS, Overall bilateral ratio, KJP 60° bilateral ratio, and LOS overall score for the first time for the professional judokas in this study.

Martial arts involve techniques such as displacement and stepping that challenge balance^37^. In previous studies, different other functional movement balance tools like star excursion and/or Y balance test scores were used to predict injuries in NCAA Division 1 athletes^23,38,39^, however considering the role of balance in judo and also the relationship between single leg stability and the risk of injury^40^, in this research, for the first time SLS, LOS, and KJP as more accurate and laboratory tools were used to check both the balance and proprioception of professional judokas of the national teams. In this research, Single leg stability and limit of stability were used using the BBS device, which is reliable for measuring postural stability^18,41,42^. Rafael Sierra-Guzmán et al. analyzed dynamic balance, peroneal reaction time and strength in athletes with Chronic Ankle Instability and healthy athletes. They achieved results that showed that the scores of Star excursion balance test and Balance test on the biodex stability system have a significant relationship with chronic ankle instability^43^. Also Trojian TH and colleagues in a prospective study on 230 male and female athletes investigated the ability of single leg balance test to predict lower limb injury. Their results also demonstrated that single leg balance is a reliable test for predicting ankle sprains^44^. In the present study, two tests of single leg stability and limit of stability were found to be suitable predictors for lower limb injury as well, and the cut point and AUC curve showed the predictive validity of these tests accurately. However, the results of the research conducted by Namazi et al. on 73 footballers showed that joint position sense has no significant relationship with the amount of lower limb injuries in footballers under the age of 21^33^. The two balance tests performed in this research are applicable in judo, because in judo the athlete tries to have fixed positions with the aim of disturbing the opponent's balance and getting points. Also, these tests were done in a closed chain just like the nature of judo^45^. Jeremy Witchalls et al. found in a meta-analysis that lower inversion JPS in women is associated with the risk of ankle injury^46^. In a study conducted by Carly May Green et al. on 40 judo players, there was no significant difference between the sense of shoulder joint position in injured and non-injured judo athletes^47^. Also, Alireza Hosseini and colleagues conducted a similar study in order to investigate the sense of proprioception and dynamic balance and its relationship with injury on 72 young elite wrestlers during 9 months. Their findings showed that these two tests are not suitable predictors for injury in young wrestlers^48^. Song et al.^49^ also demonstrated that there is a weak to moderate relationship between proprioception and dynamic and static balance control in older adults. The controversy between our findings and these studies can be addressed by the difference between the sample population and the different nature and the different anthropometry of the judokas rather than football, wrestling athletes or older adults. We can also consider the different assessment methodology addressing this contrast findings as at different knee angles, the correlation between proprioception, force sense, quadriceps muscle strength, quadriceps/hamstring ratio and balance is different^50^. It can be concluded that the lack of balance and proprioception asymmetry can cause a disturbance in a person's balance leading to making the judokas prone to sustain lower limb injuries. Finally even though the injury mechanism and anatomical position affect the injury, since these asymmetries recognized as risk factors for sustaining lower limb injuries, then sport medicine practitioners and athletic trainers are suggested to use the SLS, LOS, and KJP as pre-participation screening tools to prevent lower limb injuries in judokas^51^.

## Limitations

The current study is limited in its generalizability given small sample size and including only male participants. However, this sample included the entire population of the elite judokas of national teams in Iran (no elite female judokas were available). A formal sample size calculation was not performed for this study since all members of the national teams available in Iran were recruited (n = 53). Further recruitment would have required the addition of junior players that were not recognized as qualified target population. Furthermore, the participants selected in this research included only professional judokas and specifically the national team, and these results may be different for judokas of other levels. Future studies should also investigate female judokas. Calculating the injury exposure of each athlete may also affect the results, which was not recorded in this study. More multicenter prospective studies on a larger sample are necessary to confirm these results.

## Conclusions

The main findings of this study was that although the absolute unilateral balance and proprioception scores were almost the same between injured and non-injured judokas, the bilateral ratio of both these indices were different between the two groups. To the best of the authors’ knowledge this finding for the first time demonstrates that lower limbs bilateral balance and proprioception asymmetries is likely a more important risk factor than the absolute unilateral balance and proprioception scores for sustaining lower limb injuries in professional male judokas. This study further provided optimal cut off points of ≤ 1.26%, ≤ 1.16%, ≤ 1.15%, ≤ 1.09%, and ≥ 7.5 respectively for SLS, ML bilateral ratio, SLS, AP bilateral ratio, SLS, overall bilateral ratio, KJP 60° bilateral ratio, and LOS overall score for the first time for the national professional judokas.

## Perspectives

To the best the authors knowledge, this is the first study to investigate the screening value of the balance and proprioception indicators to predict lower limb musculoskeletal injuries in professional national level judokas. The models provided by SLS, ML bilateral ratio, SLS, AP bilateral ratio, SLS, overall bilateral ratio, KJP 60˚ bilateral ratio, and LOS overall score generally explained 12–16% of the variations of the lower limb injury (meaning that lower limb injury can be explained by these full models), suggesting that predictions by these balance and proprioception indicators are fairly reliable. Furthermore, 67.9–77.4% of the judokas were correctly classified as with lower limb injured using the models provided by these indicators which is a large improvement.

## Data Availability

The data that support the findings of this study are available from the corresponding author, MH, upon reasonable request.
